# Bone Cells Differentiation: How CFTR Mutations May Rule the Game of Stem Cells Commitment?

**DOI:** 10.3389/fcell.2021.611921

**Published:** 2021-05-07

**Authors:** Claire Dumortier, Soula Danopoulos, Frdric Velard, Denise Al Alam

**Affiliations:** ^1^Division of Neonatology, Department of Pediatrics, Lundquist Institute for Biomedical Innovation, Harbor-UCLA Medical Center, Torrance, CA, United States; ^2^Universit de Reims Champagne-Ardenne, BIOS EA 4691, Reims, France

**Keywords:** cystic fibrosis, iPSCs, bone differentiation, osteoblast, osteoclast

## Abstract

Cystic fibrosis (CF)-related bone disease has emerged as a significant comorbidity of CF and is characterized by decreased bone formation and increased bone resorption. Both osteoblast and osteoclast differentiations are impacted by cystic fibrosis transmembrane conductance regulator (CFTR) mutations. The defect of CFTR chloride channel or the loss of CFTRs ability to interact with other proteins affect several signaling pathways involved in stem cell differentiation and the commitment of these cells toward bone lineages. Specifically, TGF-, nuclear factor-kappa B (NF-B), PI3K/AKT, and MAPK/ERK signaling are disturbed by CFTR mutations, thus perturbing stem cell differentiation. High inflammation in patients changes myeloid lineage secretion, affecting both myeloid and mesenchymal differentiation. In osteoblast, Wnt signaling is impacted, resulting in consequences for both bone formation and resorption. Finally, CFTR could also have a direct role in osteoclasts resorptive function. In this review, we summarize the existing literature on the role of CFTR mutations on the commitment of induced pluripotent stem cells to bone cells.

## Introduction

Cystic fibrosis (CF) is the most common autosomal recessive genetic disorder in Caucasians, affecting 75,000 patients world-wide and occurring in approximately 1 in 3,500 newborns in the United States (Farrell et al., 2008). This disease is caused by a mutation of the cystic fibrosis transmembrane conductance regulator (*CFTR*) gene. To date, more than 2,000 mutations have been identified, resulting in the absence or dysfunction of CFTR protein: a transmembrane chloride channel, mainly known to be involved in maintaining the proper composition and amount of fluid covering different mucosal membranes in the body (Castellani et al., 2008). CFTR mutations are organized by classes based on the mechanism affected: defect of synthesis, defect of traffic through the membrane, defect of protein folding, impairment of gating, defect of conductance or defect of stability (Rowe et al., 2005). The most common mutation, accounting for approximately 80% of CF cases, is the F508del, which is a class II mutation where the protein is misfolded and destroyed before reaching the membrane. The second most common mutation, representing around 5% of total CF mutations, is the pre-mature stop mutation G542X, causing early termination of translation (Du et al., 2002). This is followed in frequency by the G551D mutation, which causes a gating defect in the CFTR channel, accounting for less than 5% of CF cases. The remaining mutations, although numerous (more than 2000), represent less than 2% of the CF mutations.

The most common and lethal manifestation of CF disease is respiratory impairment resulting from defective mucociliary clearance, bacterial infection, airway inflammation, mucus accumulation and airflow obstruction (Stoltz et al., 2015). Due to improved therapies, optimization of nutrition, and early established healthcare for children, the life expectancy of CF patients has improved significantly, thus resulting in the emergence of new comorbidities associated with the pathology: pancreatic disease and cystic fibrosis-related diabetes (CFRD), hepatobiliary disease, gastrointestinal tract, kidney disease, genitourinary disease, cystic fibrosis-related bone disease (CFBD), and coronary artery disease (Elborn et al., 2016; Ronan et al., 2017).

Cystic fibrosis-related bone disease occurs in 2035% of adults with CF, in which patients present with low bone density and osteoporosis. Prior to the evidence that CFTR dysfunction influences bone cell activity (Dif et al., 2004; Stalvey et al., 2013; Velard et al., 2014; Le Henaff et al., 2015), many of these bone defects were believed to be a result of malnutrition, sedentary lifestyle, endocrine disease, pancreatic insufficiency, delayed puberty, vitamin D and K insufficiency, calcium malabsorption, and/or use of exogenous glucocorticoids (Plant et al., 2013; Jacquot et al., 2016; Putman et al., 2019). Although it has now been established that absence or abnormal CFTR protein plays a role in bone disease, the pathways underlying the onset of CFBD remain elusive. The role of CFTR in bone cells have been determined using animal models (mice, rats, and sheep) or *in vitro* culture models using cells derived from patient biopsies. However, knowledge gained from these models remain limited due to model relevance (animal vs. human) as well as the limited accessibility to human bone biopsies from CF patients. Moreover, bone formation is initiated *in utero* and take several months to years to become a fully mature bone structure (Katsimbri, 2017). Thus, disorders resulting from CFTR absence or dysfunction may occur during embryogenesis, perturbing stem cells commitment toward bone cells. Therefore, the use of induced pluripotent stem cells (iPSCs) might represent great promise and a readily available alternative to study the effects of CFTR mutations on bone cell development. Furthermore, CF-iPSCs and healthy iPSCs, *via* CRISPR/Cas9-mediated correction of the *CFTR* gene, can be generated and compared from the same patient, providing controls with identical genetic background.

Induced pluripotent stem cells provide an opportunity to develop any cell type from an easily accessible somatic cell source. Human iPSCs can be generated from a wide spectrum of somatic cells, including fibroblasts, keratinocytes, mesenchymal stem cells (MSCs) or peripheral blood mononuclear cells (Abdal Dayem et al., 2019). Pluripotency is induced with a combination of reprogramming factors: OCT3/4, SOX2, KLF4, L-MYC, LIN28, and shRNA for TP53 (Okita et al., 2013). iPSCs have normal karyotypes, maintain telomerase activity, express characteristic cell surface markers and genes of human embryonic stem cells (ESCs), possess high self-renewal capacity and maintain the developmental potential to differentiate into mature cells of all three primary germ layers (Yu et al., 2007).

This review explores the main pathways involved in osteoblast and osteoclast differentiation from iPSCs and summarizes which pathways are known to be impacted by CFTR absence or malfunction.

## iPSCs Commitment Toward Mesenchymal Stem Cells and Hematopoietic Lineage

Bone cells derive from hematopoietic and mesenchymal precursor cells. From the undifferentiated pluripotent stage until terminal differentiation forming osteoblasts, osteocytes and osteoclasts, the two lineages are related. Sacchetti et al. (2007) proved the self-renewal potential of osteoprogenitors in bone marrow sinusoids by showing their capacity to organize the hematopoietic microenvironment, suggesting the link between MSCs and myeloid ones. MSCs express angiogenic proteins, allowing the formation of cartilage, which in turn induce a better angiogenesis. On the other hand, monocytes or osteoclast precursors can differentiate into mature osteoclasts. Osteoclast resorption along with matrix mineralization promote osteogenic differentiation, which in turn regulate the osteoclast commitment and bone resorption (LoGuidice et al., 2016). Myeloid and lymphoid cells circulating in blood and MSCs in bone marrow secrete factors essential for stem cell renewal or differentiation, osteoblasts and osteoclasts differentiation thus regulating bone formation or resorption. Finally, the mature bone cells also secrete components of the different pathways allowing bone modeling and remodeling.

### Generation and Characterization of MSCs and Hematopoietic Lineage From iPSCs

Mesenchymal stem cells are pluripotent cells able to differentiate into a variety of mature cell types: adipocytes, myocytes, chondrocytes, and osteoblasts. The differentiation potential of iPSCs into functional MSCs can be achieved using different methods: use of growth factors [basic fibroblast growth factor (bFGF), epidermal growth factor (EGF), and platelet-derived growth factor (PDGF-/)] in combination with CD24/CD105+ sorting, repeated passage with trypsinization, culture in hypoxic condition with growth factors, embryoid bodies (EBs) formation, biomimetic, fibrillar, type I collagen coatings, and use of small molecule inhibitors [such as transforming growth factor- (TGF-) pathway inhibitor)] (Abdal Dayem et al., 2019). However, each method presents with its own set of advantages and disadvantages such as time-consuming laborious techniques, efficacy of differentiation and tumorigenicity of iPSC-induced cells (Abdal Dayem et al., 2019). The International Society for Cellular Therapy (ISCT) proposed the following basic criteria to characterize MSCs (Dominici et al., 2006): exhibition of typical fibroblastic cell morphology, expression of MSC surface markers CD44, CD73, and CD105, and the potential to differentiate into the three different cell lineages: osteoblasts, chondrocytes and adipocytes.

Different protocols have been used to differentiate iPSCs into myeloid cells, pre-requisite step to osteoclast commitment. Basically, three differentiation methods are described: co-culturing iPSCs with stromal cells, EBs formation, and monolayer cultures of iPSCs on extracellular matrix protein coated plates, such as collagen IV (Chen, 2014). The different protocols with their advantages and disadvantages were reviewed by Chen (2014).

Early mesoderm formation is indicated by the expression of *Brachyury* (*TBXT*), *MIXL1*, and *GSC* (Herrmann, 1991) in conjunction with pluripotency genes silencing. In primitive hematopoietic precursors, co-expression of *MIXL1* and *PDGFRA* genes is highly enriched (Davis et al., 2008). The final characterization of myeloid progenitors is represented by Lin-CD34+CD43+CD45+ cell population (Choi et al., 2009).

Different transcription factors are required for hematopoietic commitment. Hemangioblasts are the first differentiated mesodermal derivatives, displaying both hematopoietic and endothelial potential. Runt-related transcription factor 1 (RUNX1) is important for the hemangioblast stage and erythroid lineage commitment is RUNX1-dependent (Lacaud, 2002). In the absence of stem cell leukemia/T-cell acute lymphoblastic leukemia 1 (SCL/tal-1) transcription factor, hematopoiesis is undetectable (Porcher et al., 1996). However, SCL/tal-1 expression may be induced by the addition of bone morphogenetic protein 4 (BMP4) and VEGF. Furthermore, VEGF-R2, which is already detected in human iPSCs, is increased during mesoderm to hematopoietic lineage transition (Kennedy et al., 2007). Finally, GATA1 and GATA2 transcription factors have also been shown to be involved in hematopoietic commitment (Iwasaki et al., 2003).

### The TGF- Signaling Pathway

Members of the TGF- superfamily, which includes TGF-, bone morphogenetic proteins (BMPs), activins, and growth and differentiation factors (GDFs), are secreted proteins that have important roles in directing mesenchymal cell fate. By binding to transmembrane receptors with serine/threonine kinase activity (type I and type II also called activin receptor-like kinases, ALKs), the TGF- family members initiate intracellular signaling through phosphorylation of specific SMAD proteins, which in turn translocate from the cytoplasm to the nucleus and control the transcription of target genes (Roelen and ten Dijke, 2003).

**FIGURE 1:**
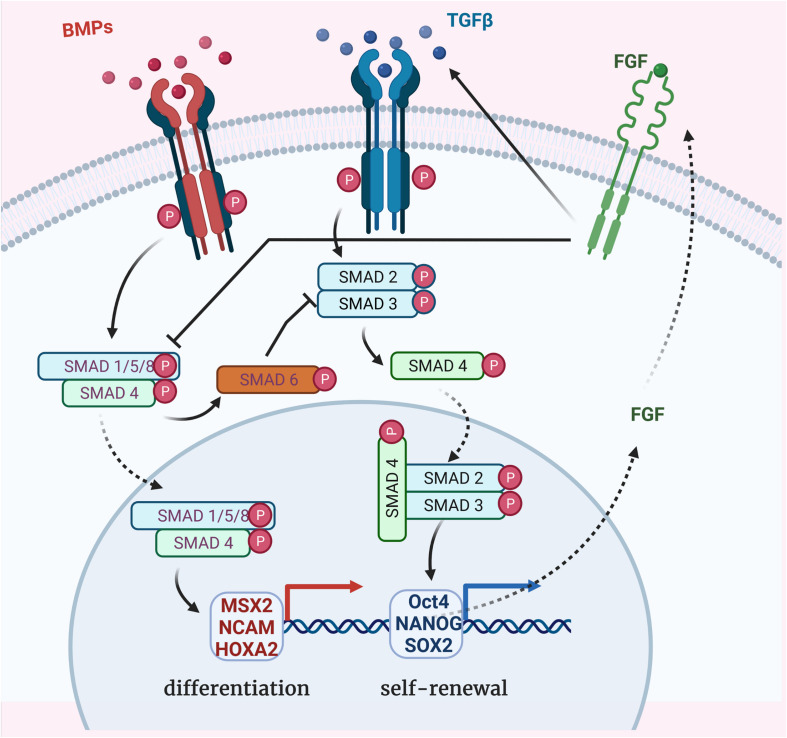
Bone morphogenetic protein (BMP), TGF-, and FGF-dependent SMADs pathways for regulation of stem cell differentiation. TGF- super-family members binding to the receptors which propagate phosphorylation signal to receptor-regulated SMAD proteins (R-SMADs in blue). BMP signaling occurs through SMADs 1/5/8 phosphorylation and TGF- signaling through SMADs 2/3 phosphorylation. Once activated, their binding with the common partner (Co-SMAD in green) SMAD 4 results in complexes which translocate to the nucleus to activate other transcription factors and regulate specific genes expression. MSX2, NCAM, and HOXA2 expression leads to the differentiation of ESCs whereas Oct4, NANOG, and SOX2 expression contributes to the undifferentiated proliferation. BMP signaling negatively regulates TGF- signaling via the expression of the inhibitory SMAD 6 (I-SMAD in orange). FGF signaling promotes TGF- receptor (TGFBR1) activation, resulting in self-renewal transcription factors activation and, in turn, FGF expression. FGF signaling also directly represses SMAD1/5/8 phosphorylation, inhibiting the BMP pathway.

Transforming growth factor- family has been shown to preserve cell morphology of undifferentiated ESCs by maintaining *POU5f1*, *NANOG*, *TRA-1-60*, and *SSEA4* expression, through increased phosphorylation of SMADs 2/3 (Hannan et al., 2009). Conversely, inhibition of TGF- reduces SMADs 2/3 phosphorylation in ESCs resulting in the loss of ESC phenotype and pluripotency (Snchez et al., 2011). TGF- signaling (through SMAD-2/3) negatively regulates MSC generation from human ESCs. Alternatively BMP signaling promotes ESC differentiation by activating SMADs 1/5/8 (Xu et al., 2002; Figure 1).

Interestingly, inhibiting FGF receptor 1 (FGFR1) increases MSC differentiation without affecting cell number, apoptosis or cell cycle status, suggesting that FGF signaling plays a role in ESCs maintenance (Bendall et al., 2007). Fibroblast growth factor 2 (FGF2) is known to promote self-renewal of human ESCs by modulating the expression of TGF- ligands: TGF-1, GREM1 (a BMP antagonist), and BMP4 (Greber et al., 2007). Furthermore, FGF signaling interacts with the TGF- pathway to synergistically inhibit BMP signaling, directly by repressing SMAD1/5/8 phosphorylation or indirectly by promoting SMAD2/3 phosphorylation, allowing for the maintained expression of pluripotency genes (NANOG, OCT4, and SOX2) and promoting long-term undifferentiated proliferation of human ESCs (Xu et al., 2008). In summary, self-renewal of human ESCs is promoted by TGF- signaling, whereas differentiation is promoted by TGF- inhibition or BMP signaling.

However, iPSCs and ESCs differ in their commitment toward MSCs, whereby iPSCs pose a greater challenge due to their resistance to SMADs 2/3 inhibition (Snchez et al., 2011). To overcome this challenge, a TGF- pathway inhibitor SB-431542 is added to serum-free medium, resulting in MSC differentiation from human iPSCs (Chen Y. S. et al., 2012). After 10 days, iPSCs showed downregulation of pluripotency genes and upregulation of mesodermal genes (*MSX2*, *NCAM*, and *HOXA2*), thus proving that TGF- pathway inhibition is an efficient method for the commitment of iPSCs toward MSCs.

### Other Signaling Pathways Known to Maintain Pluripotency

Other pathways such as MAPK/ERK, PI3K/AKT, and NF-B are necessary to maintain the pluripotent and undifferentiated state of ESCs (Armstrong et al., 2006).

The RAS-Mitogen Activated Protein Kinase (MAPK) pathway transduces signals from cytokines and growth factors through Receptor Tyrosine Kinases (RTK), causing ERK1/2 to translocate to the nucleus and activate JUN and FOS transcription factors. This pathway could be activated by FGF ligands, also involved in the TGF- pathway. MAPK/ERK pathway is active in undifferentiated human ESCs and upon differentiation, several components of this pathway are downregulated such as RASAL2, SOS1, RAF, MAP2K6, or KRAS (Armstrong et al., 2006).

The PI3K/AKT pathway is activated by cytokines and growth factors, but also endogenously by Ras family protein Eras (ES cell-expressed Ras) (Dreesen and Brivanlou, 2007). Phosphoinositide 3-kinase (PI3K) phosphorylates PIP2 (phosphatidylinositol-4,5-bisphosphate), converting it to PIP3 (phosphatidylinositol-3,4,5-trisphosphate). This can be reversed by the Phosphatase and tensin homologue (PTEN). One major downstream mediator of PIP3 is AKT which is activated by Pyruvate Dehydrogenase Kinase 1 (PDK1). Phosphorylated AKT then regulates a number of downstream targets. PI3K is important for the maintenance of undifferentiated murine ESCs and promotes short-term self-renewal (Paling et al., 2004). In fact, it has been reported that tumor suppressor p53 promoted differentiation of murine ESCs by suppressing *NANOG* expression. However, this is dependent on the phosphorylation of Ser315 of p53 which is a residue substrate of Glycogen Synthase Kinase 3 Beta (GSK3). And GSK3 is negatively regulated by PI3K and AKT (Lin et al., 2005).

The NF-B transcription factor family consists of p50/p105, p52/p100, c-Rel, RelA (also known as p65), and RelB, which altogether regulate the expression of hundreds of target genes. In the absence of signaling, these factors are inactivated by the interaction with IB inhibitory protein. NF-B pathway is activated by a variety of extracellular factors such as tumor necrosis factor alpha (TNF), interleukin 1 (IL1), growth factors, bacterial or viral infections or oxidative stress. In response to such stimuli, IB is phosphorylated, ubiquitinated and degraded, allowing the NF-B factors to freely translocate to the nucleus. A number of NF-B components have been shown to decrease upon cell differentiation, such as LCK, a lymphocyte-specific tyrosine kinase, and PELLINO1 (both required for NF-B activation) as well as TNFSF11/RANKL, the receptor activator of NF-B ligands (Armstrong et al., 2006). Moreover, RelA is only present in the nucleus of undifferentiated ESCs, indicating active NF-B pathway. When miRNA was used to target p65/RelA, ESC pluripotency was lost, resulting in epithelial to mesenchymal transition (Lningschrr et al., 2012). Finally, inhibition of NF-B signaling enhances the differentiation of human ESCs into MSCs by diminishing expression of pluripotent markers and increasing the expression of MSC surface markers. The depletion of p65 led to a 3-fold increase of CD73+CD90+CD146+CD45 MSCs (Deng et al., 2016).

**FIGURE 2:**
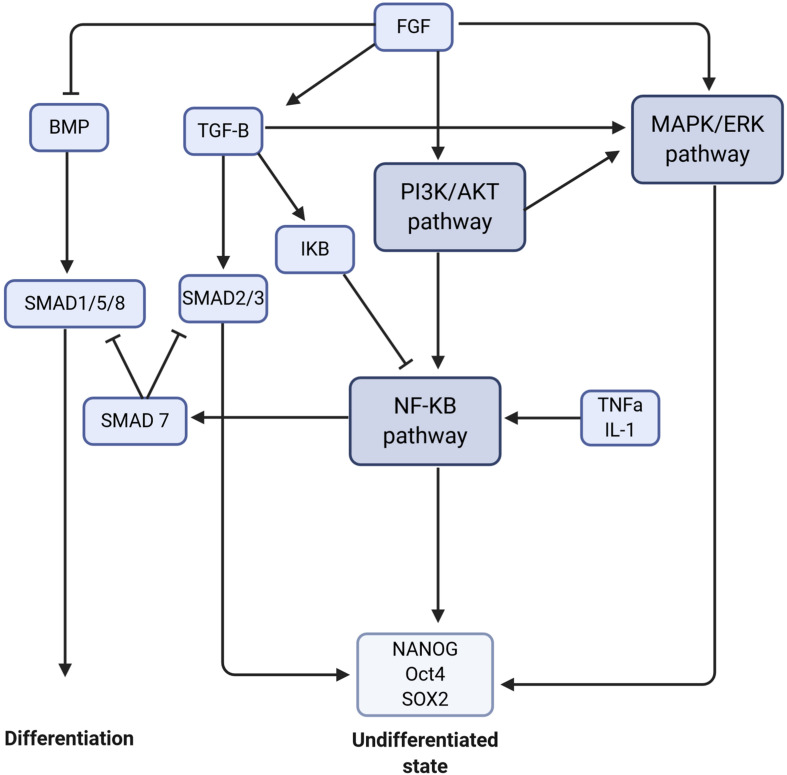
Crosstalk between signaling pathways to maintain stem cells in an undifferentiated state or to trigger the differentiation. The signaling pathways are in dark purple, the molecules/proteins are represented in blue, and the transcription factors in light blue. The arrows mean activation and the flat arrows inhibition.

Crosstalk amongst these pathways have been described in numerous cell processes. The receptor activator of NF-B ligand (RANKL/TNFS11) has been shown to be translationally regulated by PDK1 of the PI3K/AKT pathway (Tanaka et al., 2005). PDK1 activates NF-B signaling by phosphorylating and degrading IB, thus allowing p65 to enter the nucleus. Figure 2 shows the interaction between these different pathways to maintain the ESCs in an undifferentiated state.

To summarize, the different protocols for iPSC commitment to MSCs are based on the main pathways described above. Various growth factors or small molecule inhibitors can be used to either activate or inhibit of a specific pathways, which in turn may activate or inhibit others.

## Osteogenic Commitment and Bone Formation

Mesenchymal stem cells express *RUNX2* (transcription factor required for osteoblast cell fate), *SOX 9* (chondrocyte-specific transcription factor), and *PPARG* (adipocyte-specific transcription factor) and have the ability to differentiate into one of three cell-types: osteoblasts, chondrocytes, or adipocytes. This is determined by the different genetic pathways expressed in MSCs. For osteogenic differentiation, cells must go through three different stages: (1) the immature osteoblasts with *COL1A1*, *ALPL*, and *SSP1* differentiation markers (respectively collagen 1, alkaline phosphatase, and osteopontin genes), (2) the mature osteoblasts which express *BGLAP* (osteocalcin gene), and (3) terminal differentiation into osteocytes with *SOST* (sclerostin gene) and Dentin matrix acidic phosphoprotein 1 (*DMP-1*) expression (Ducy et al., 1997).

Osteoblast differentiation from MSCs is primarily dependent on the BMPs, parathyroid hormone (PTH) and Wnt pathways.

### The Bone Morphogenetic Proteins Pathway

Bone morphogenetic proteins are known to activate both the SMAD and MAPK pathways. In the SMAD pathway, both type I and type II BMP receptors are needed for signal transduction. Upon the activation of these receptors by BMP ligands such as BMP-2 and BMP-4, the intracellular SMADs 1, 5, and 8 become phosphorylated and form a complex with SMAD 4. This complex translocates into the nucleus and participates in gene transcription (Figure 1). SMADs 1 and 5 directly interact with the bone-specific transcription factor RUNX2 and activate the transcription of target genes such as *COX-2* and *COL10A1* in osteoblasts and chondrocytes (Zhao et al., 2003). SMAD1 directly interacts with HOWC8 protein to promote osteopontin production (Yang et al., 2000). Moreover, both SMAD1 and RUNX2 undergo ubiquitin-proteasome-mediated degradation. SMURF1 (SMAD Specific E3 Ubiquitin Protein Ligase 1), a member of the HECT family of E3 ubiquitin ligase, has been found to interact with SMADs 1 and 5, thereby triggering their ubiquitination and degradation. Inhibition of SMURF1 and proteasome degradation lead to increased osteoblast function and bone formation (Zhao et al., 2003).

For the MAPK pathway, BMP-2 has been shown to activate ERK1/2, p38 and JNK in human osteoblastic cells, inducing osteoblast differentiation with the increased expression of alkaline phosphatase (ALP) and osteocalcin (Guicheux et al., 2003). In addition, BMP-2-activated-ERK1/2 inhibits collagen X expression in osteoblasts, resulting in increased phosphorylation of RUNX2, which in turn upregulates ALP expression (Reilly et al., 2005).

### Influence of Parathyroid Hormone (PTH) on Osteoblasts

Parathyroid hormone is the primary calcium metabolism regulating hormone. Osteoblasts are rich in PTH receptors and PTH-related protein receptors (Gardinier et al., 2019). Intermittent PTH injections in rat promotes osteoblast differentiation and bone formation, whereas continuous PTH injection inhibits osteogenesis (Frolik et al., 2003). It has been explained that continued exposure to PTH *in vitro* causes a desensitization of the adenylate cyclase and phospholipase C responses as well as receptor downregulation (Frolik et al., 2003). It was further confirmed in human that levels of PTH expression and PTH receptors influence osteoblast formation (Osagie-Clouard et al., 2017). This can be explained by several molecular pathways.

First, PTH promotes ubiquitinylation, ultimately stimulating proteasome activities resulting in the degradation of osteoblast protein substrates (Murray et al., 1998). Moreover, the anabolic effect of PTH is RUNX2-dependent (Krishnan et al., 2003). The binding of PTH to PTH receptor 1 (PTH1R) stimulates production of cAMP and activation of protein kinase A (PKA). PKA subsequently phosphorylates transcription factors such as RUNX2 and c-AMP-response element-binding protein (CREB) thus promoting intracellular free Ca^2+^ which in turn regulates TGF-1 expression (Wu et al., 2009).

**FIGURE 3:**
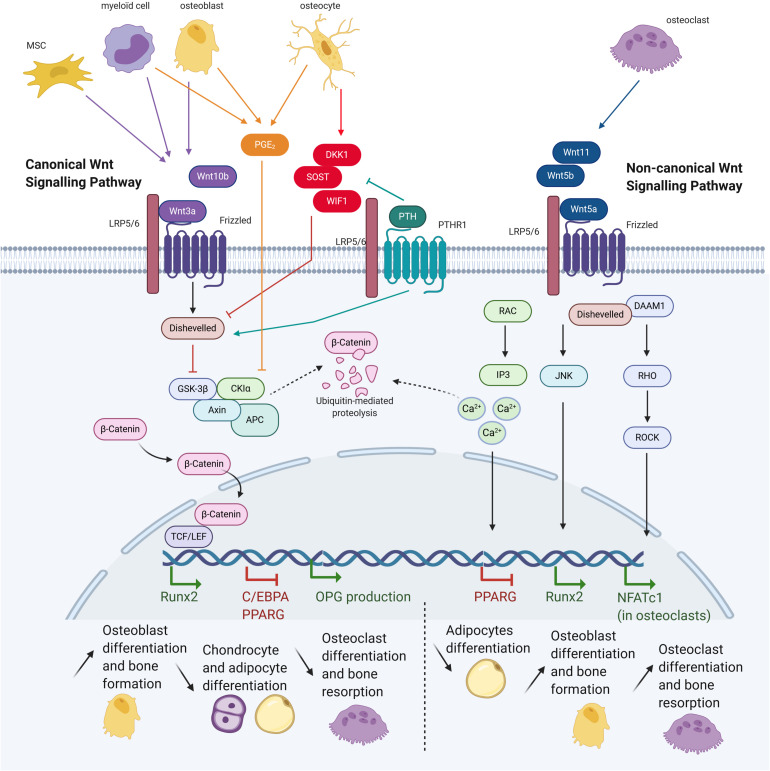
Wnt pathway in osteoblast and consequences on bone cells differentiation. Mesenchymal stem cells (MSCs), myeloid cells, osteoblasts, osteoclasts, and osteocytes express different molecules, triggering or inhibiting the canonical or non-canonical Wnt signalings. Both signalings allow translation of different genes, having consequences for cells commitment and bone activity (Adapted from Baron and Kneissel, 2013).

Parathyroid hormone is also involved in the Wnt pathway (Figure 3). Mice expressing constitutively active PTH1R in osteocytes present increased Wnt signaling and bone mass, whereas deletion of the co-receptor LRP5 suppresses this bone gain (OBrien et al., 2008). Conversely, mice lacking PTH1R in osteocytes demonstrate osteopenia associated with increased *SOST* expression and decreased canonical Wnt signaling (Powell et al., 2011). Furthermore, PTH1R has been shown to activate the Wnt pathway in the absence of Wnt ligands by forming a complex with LRP5/6 after PTH binding. This leads to the phosphorylation of LRP6 which allows for the recruitment of axin and -catenin stabilization (Wan et al., 2008). PTH also represses expression of several secreted Wnt antagonists, such as Sost, DKK1, and Wif1 (Li et al., 2007; Guo et al., 2010).

Finally, PTH has been shown to activate PI3K/AKT signaling by phosphorylating AKT (Yamamoto et al., 2007). AKT phosphorylation then promotes Signal transducer and activator of transcription 5 (STAT5) phosphorylation resulting in increased receptor activator of nuclear factor kappa-B ligand (RANKL) expression (Tsubaki et al., 2007). In turn, RANKL upregulation stimulates osteoclast precursor commitment into mature osteoclasts.

In summary, PTH is involved in several molecular pathways that regulate osteoblast differentiation, bone formation, and/or bone resorption.

### The Wnt Pathway

#### The Canonical Pathway

Osteoblasts are located close to the bone marrow, which serves as a major source for MSCs and hematopoietic stem cells (Spradling et al., 2001). Osteoblasts and peripheral blood mononuclear cells (PBMCs) are involved in the regulation of hematopoietic stem cells by releasing many factors that impact differentiation, such as Wnt family proteins. Some Wnt secreted glycoproteins, like Wnt3a and Wnt10b, can bind to a Frizzled receptor (FZD) and recruit the Low-density lipoprotein receptor-related proteins 5/6 (LRP5/6) co-receptors, thus activating the canonical signaling pathway (Figure 3). The proteolysis GSK-3 complex is inhibited by activating Dishevelled (Dsh) protein, resulting in -catenin stabilization and translocation into the nucleus. This then regulates the T-cell factor/lymphoid enhancer factor (TCF/LEF) transcriptional activity which in turn activates downstream genes. Canonical Wnt signaling can be inhibited by osteocyte secreted factors such as sclerostine, Dickkopf WNT Signaling Pathway Inhibitor 1 (DKK1) or Secreted Frizzled Related Protein 1 (SFRP1), which bind to LRP5/6 causing its inactivation. In result, GSK-3 is no longer inhibited and can target -catenin to be ubiquitinated and degraded (Logan and Nusse, 2004).

Canonical Wnt activity is essential for the development and differentiation of multiple organ systems including bone. Canonical Wnt signaling enhances ossification and suppresses chondrocyte formation, whereas inhibition of -catenin in MSCs results in chondrocyte differentiation (Day et al., 2005). Furthermore, Wnt10b, which is expressed in bone marrow by osteoblast progenitors (Andrade et al., 2008) and T lymphocytes (Terauchi et al., 2009), stimulates osteoblastogenesis and inhibits adipogenesis of mesenchymal precursors (Bennett et al., 2005). Osteogenic commitment was confirmed by upregulation of osteoblastogenic transcription factors RUNX2, DLX5, and OSTERIX, whereas inhibited adipocyte differentiation was validated by suppression of the adipogenic transcription factors C/EBPA and PPARG. In contrast, deficiency in the canonical Wnt inhibitor DKK1 was associated with increased bone formation in mice and humans whereas its presence in mesenchymal progenitor cells lead to adipocyte commitment (Pinzone et al., 2009). Using RNAi to knock-down LRP5 and -catenin expression, it was shown that only *ALPL* seemed to be positively regulated by the canonical Wnt pathway, whereas the other osteogenic markers were negatively affected (Ilmer et al., 2009).

Conversely, other studies showed opposing effects where Wnt3a inhibits *in vitro* MSC osteogenic differentiation, with decreased matrix mineralization and reduced ALP mRNA levels and activity (Boland et al., 2004). This was corroborated by other reports demonstrating that Wnt3a and LRP5 overexpression inhibited osteogenic differentiation (Baksh et al., 2007), and that -catenin inhibits osteoblast differentiation of human MSCs *in vitro* in osteogenic medium (Zhou, 2011). This controversy into the role of canonical Wnt signaling on osteogenic differentiation from MSCs was resolved by Liu et al. (2009) who argued that under conditions permissive for binary lineage differentiation, Wnt signaling could shift the commitment from adipocytes toward osteoblasts, whereas osteoblast differentiation remains inhibited in osteogenic conditions. Others reported that Wnt10b induction of osteogenesis in mouse progenitors was due to inhibition of *PPARG* and *C/EBPA* activity (Kang et al., 2007).

The common osteoblast differentiation medium contains dexamethasone, ascorbic acid and -glycerophosphate. These three compounds play a critical role in the different pathways required for osteoblast differentiation (Langenbach and Handschel, 2013). Dexamethasone activates Wnt/-catenin signaling by upregulating FHL2, a LIM-domain protein which in the presence of Wnt3a potentiates -catenin transport to the nucleus (Hamidouche et al., 2008). The addition of Ascorbic acid and -glycerophosphate facilitates osteogenic differentiation by enhancing Runx2 activity via the MAPK signaling pathway (Xiao et al., 2002).

Canonical Wnt signaling also indirectly represses osteoclast differentiation and bone resorption through the increased secretion of osteoprotegerin (OPG), a major inhibitor of osteoclast differentiation. Its expression is regulated by -catenin and TCF proteins (Glass et al., 2005). In addition, Wnt3a also inhibits murine osteoclast differentiation (Santiago et al., 2012).

Other pathways that regulate GSK-3 may also modulate the canonical Wnt pathway. Activation of AKT or integrin-like kinases (ILK) has been shown to upregulate -catenin level by inhibiting GSK-3 through phosphorylation (Topol et al., 2003). Coordination between TGF- and canonical Wnt signaling was shown to promote chondroblast differentiation at the expense of adipocytes and osteoblasts (Zhou, 2011). TGF-1 activates -catenin signaling pathway *via* ALK-5, SMAD3 [which prevents -catenin degradation and facilitates its nuclear translocation (Jian et al., 2006)], SMAD4 [which interacts with -catenin and TCF/LEF1 (Nishita et al., 2000)], PKA and PI3K pathways and requires ALK5, PKA, and JNK interaction to inhibit osteoblastogenesis in human MSCs.

#### The Non-canonical Pathway

The non-canonical Wnt pathway is activated by other Wnt proteins such as Wnt-5a, -5b, and -11, and is mediated *via* Rho-GTPase-proteins, calcium fluxes and/or c-Jun N-terminal kinases (JNK) (Figure 3). Non-canonical Wnt ligands bind to FZD and recruit Dsh protein to form the DAAM1 complex. This then triggers activation of the small G protein RHO, in turn activating RHO-associated kinase (ROCK), leading to the inhibition of NFATC1 transcription factor in osteoclasts. Alternatively, Dsh may also form a complex with RAC, resulting in JNK activity and the activation of *RUNX2*. Finally, the Wnt-Ca^2+^ pathway is activated by osteoblast-expressed Wnt-5a binding to FZD and to the osteoclast-expressed co-receptor ROR2. Intracellular calcium concentrations increase, resulting in dystoglycan 1 (DAG) and inositol 1,4,5-triphosphate, type 3 (IP3) generation. cGMP amount decreases, causing the inhibition of *PPARG* and pro-osteogenic commitment (Bilkovski et al., 2010).

The osteoclast differentiation was also achieved by this Wnt-5a-Ror2 non-canonical pathway (Maeda et al., 2012) and in turn osteoclasts release Wnt ligands to trigger osteoblastogenesis. However, releasing intracellular calcium may also activate protein kinase C which can antagonize the canonical pathway by promoting degradation of -catenin (Topol et al., 2003). Lastly, the non-canonical Wnt4 protein improves osteogenic differentiation *in vitro* and promotes bone regeneration and repair *in vivo* (Chang et al., 2007).

In summary, the canonical Wnt pathway allows osteoblast commitment of MSCs by inhibiting adipocyte and chondrocyte differentiation, promotes bone formation and represses bone resorption. In contrast, the non-canonical Wnt pathway induces both bone formation and bone resorption *via* osteoclastogenesis.

### Effect of CFTR Mutations on Canonical Wnt Pathway

Cystic fibrosis transmembrane conductance regulator is an ATP-binding cassette (ABC) transmembrane chloride channel that belongs to the ABC transporters family. It is composed of two repeated motifs: six hydrophobic membrane helices and a cytoplasmic hydrophilic region for ATP binding. These motifs are linked by a cytoplasmic regulatory domain that possesses many phosphorylation sites (Riordan et al., 1989). The terminal tails, located in the cytoplasm, mediate several interactions with binding proteins. The carboxyl terminus contains a PDZ-binding domain (PDZBD), which can bind to other proteins with PDZ domain (Li and Naren, 2011). CFTR is expressed in osteoblasts and osteoclasts, but in lower amount than in epithelial cells (Le Henaff et al., 2016).

**FIGURE 4:**
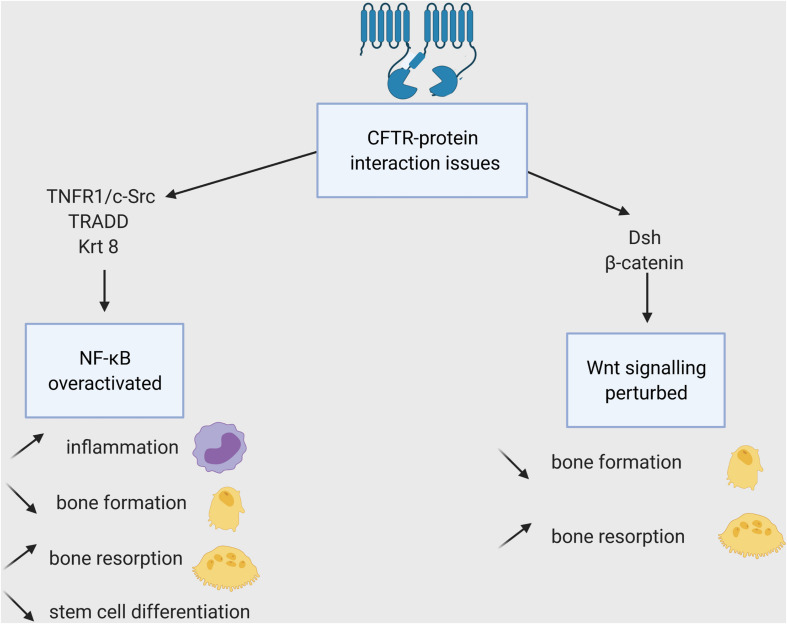
Impacts of the CFTR-proteins interactions loss on bone cells. WT-CFTR, because of its PDZBD interacts with c-Src, TNFR1 and TRADD (shown in epithelial cells), Krt8 (in osteoblasts), and Dsh (in osteoblasts, osteoclasts, and osteocytes). The lack of these interactions leads to NF-KB overexpression and Wnt signaling disruption and to several consequences for bone cells and their progenitors.

**FIGURE 5:**
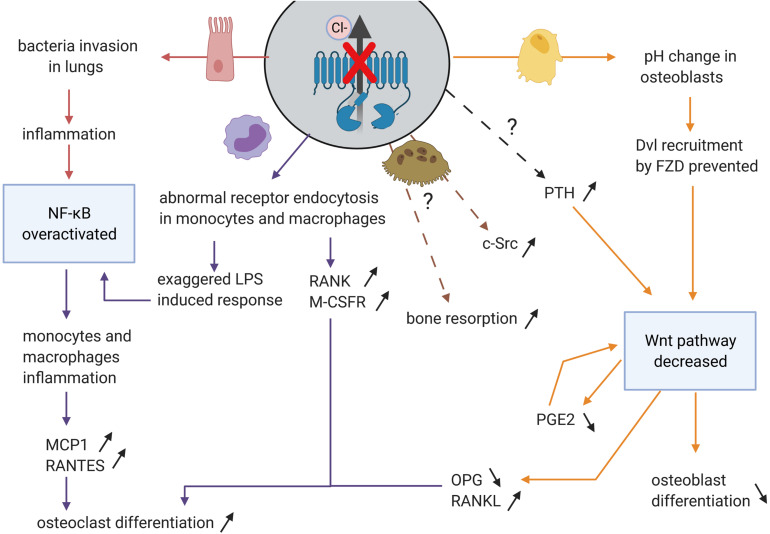
Impact on disruption of chloride channel function of CFTR in the different organs and the consequences for osteoblasts and osteoclasts differentiation and activity. CFTR channel function defect impacts airway epithelial cells (in pink), monocytes and macrophages (in purple), possibly directly osteoclasts (in brown), and osteoblasts (in yellow). In these cells, some pathways (in blue) are disturbed leading to increased osteoclast differentiation and bone resorption and decreased osteoblast differentiation and bone formation.

Loss of CFTR protein or/and its chloride channel function has a detrimental impact on bone cells. Recent studies in CFTR-deficient new-born pigs showed high porosity of cortical bones and altered chemical composition of the trabecular bones (Braux et al., 2020). *Cftr*/ mice display severe osteopenia in both trabecular and cortical bone (Dif et al., 2004). They demonstrate drastic reduction in bone formation accompanied by increased bone resorption. Delayed osteoblast differentiation, reduced ALP expression and increased proliferative rate were shown in F508del CFTR bone marrow stromal cells (Velard et al., 2014; Le Henaff et al., 2015). The main outcomes resulting from CFTR mutations are its diminished ability to interact with other proteins (Figure 4) and faulty chloride channel function (Figure 5), both ultimately influencing bone cell differentiation.

Canonical Wnt signaling is impacted by these two outcomes. Dsh protein contains a PDZ domain. In zebrafish, it has been shown that CFTR interacts with Dsh *via* PDZBD regardless of its channel function (Sun et al., 2018). The use of CFTR PDZBD deletion suggested the importance of CFTR in stabilizing Dsh through a direct protein-protein interaction. Moreover, CFTR deficiency resulted in accelerated Dpr1-induced lysosomal degradation of Dsh by preventing the Dpr1-Dsh interaction. The impaired Wnt signaling was rescued by overexpression of G551D CFTR mutant, which causes a chloride channel defect but keeps the PDZBD domain intact. Moreover, the lack of CFTR channel function was also directly involved since Wnt pathway is pH- and charge-dependent (Simons et al., 2009). The change in pH induced by defective CFTR, resulting in increased extracellular acidification (Massey et al., 2021), perturbs the recruitment of Dsh by FZD, which does not possess any specific binding site but uses some polybasic amino acid to interact with acidic and negatively charged lipids of the plasma membrane (Wong et al., 2000). Furthermore, studies have shown that CFTR directly interacts with -catenin to regulate the differentiation of ESCs into mesoderm, a critical step toward bone lineage differentiation (Gadue et al., 2006; Liu et al., 2017). Such direct interaction of CFTR with -catenin might therefore play an important role in bone lineage commitment and differentiation.

Finally, it has been shown that Cftr/ mice have increased level of PTH (Dif et al., 2004). As previously described, PTH has an ambivalent role in bone development, being an activator of the canonical Wnt signaling pathway and permitting bone formation, as well as inhibiting osteogenesis. Therefore, it is plausible that CFTR mutations alter bone differentiation through the interference with the canonical Wnt pathway and PTH.

## Osteoclast Differentiation and Bone Resorption

### The RANKL/GM-CSF/MCP-1/RANTES Pathway

**FIGURE 6:**
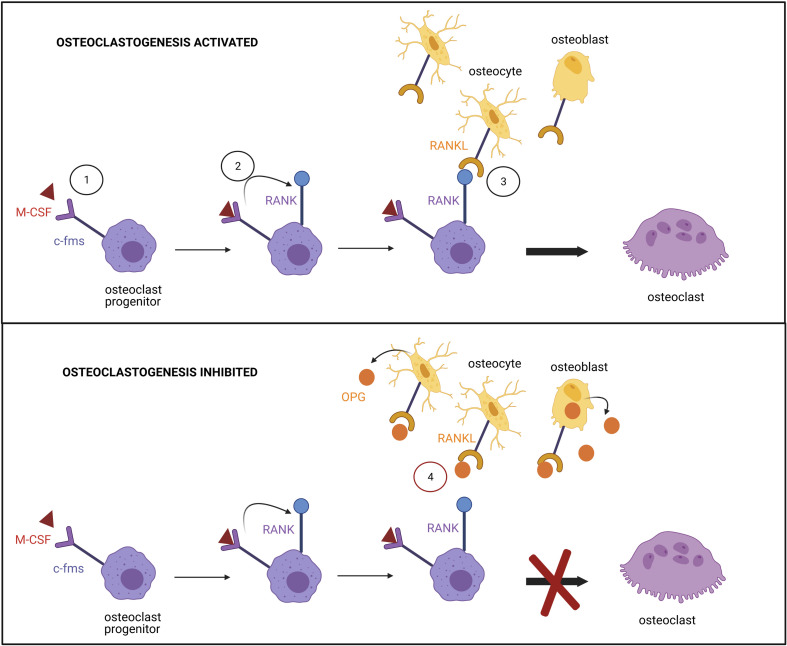
Regulation of the osteoclast differentiation. (1) Binding of M-CSF to its receptor c-fms at the surface of osteoclast progenitor promotes (2) RANK expression. (3) Signaling induced by the binding of RANK to RANKL, expressed by osteocytes and osteoblasts, triggers transcription factor activation and osteoclast differentiation. (4) The differentiation is prevented by the high affinity association of osteoprotegerin (OPG), produced by osteoblasts, to RANKL.

Osteoclasts are multinucleated cells that express tartrate-resistant acid phosphatase (TRAP) and have a bone resorption function. They are differentiated from mononuclear precursor cells of the monocyte macrophage lineage. RANKL and macrophage colony-stimulating factor (M-CSF) are crucial cytokines for osteoclast differentiation (Yoshida et al., 1990). RANKL, expressed at the surface of osteocytes and osteoblasts, interacts with RANK on osteoclast precursors, resulting in a cascade of gene expression controlled by transcription factors including NF-B and NFATC1. The differentiation depends on signaling through c-fms (the receptor for M-CSF) in mononuclear precursor cells which in turn up-regulate RANK expression (Figure 6). Proximity between osteoblastic lineage and hematopoietic cells is therefore required to form osteoclasts. The two factors induce expression of osteoclast marker genes such as *ACP5*, *CTSK*, *CALCR*, *TRAP*, and *ITGB3* (Boyle et al., 2003).

Osteoclast differentiation is regulated by OPG (a soluble antagonist of RANKL also secreted by the osteocytes), which binds to RANKL with high affinity and inhibits its action. The OPG/RANKL ratio determines the degree of osteoclast differentiation (Hofbauer and Heufelder, 2001). T and B cells produce several cytokines to regulate osteoclast differentiation. Granulocyte macrophage colony-stimulating factor (GM-CSF), secreted by T cells, has shown both inhibitory and stimulatory effects on osteoclast formation. It has been reported that short-term treatment triggers osteoclast differentiation, whereas long-term exposure suppresses it (Hodge et al., 2003). Monocyte chemotactic protein-1 (MCP-1), a cytokine expressed by mature osteoclasts, is regulated by NF-B (Donadelli et al., 2000). Furthermore, RANKL-induced NFATC1 signaling has been demonstrated to increase expression of MCP-1 and RANTES chemokine (Regulated upon Activation Normal T cell Expressed and Secreted), promoting the formation of TRAP-positive multinuclear and bone-resorptive cells (Kim et al., 2005). The addition of exogenous MCP-1 reverses the GM-CSF mediated suppression of osteoclast formation, permitting bone resorption. Consequently, it has been suggested that pathologies associated with high levels of MCP-1 will result in increased osteoclast differentiation and bone resorption.

### Regulation of Resorptive Function of Osteoclasts by Other Cells

There are a number of signaling pathways that influence bone resorption. Downregulation of NF-B transcription factors results in diminished osteoclastogenesis which causes osteoporosis (Franzoso et al., 1997). As previously described, TGF- inhibits the NF-B pathway by increasing IB inhibitors, whereas TNF, IL1, or FGF through PI3K/AKT activate it.

The canonical Wnt pathway also influences bone resorption *via* PTH or prostaglandin E2 (PGE2). PGE2, secreted by osteoblasts and osteocytes (Rochefort et al., 2010), is essential for Wnt signaling in stem cells, promoting the differentiation of several lineages by interacting with the -catenin destruction complex (Goessling et al., 2009). PGE2 is also expressed in cells of the hematopoietic lineage (Hackett et al., 2006), directly affecting osteoclastic cells. Interestingly, whereas PGE2 increases RANKL-stimulated osteoclast differentiation in murine cells (Kobayashi et al., 2005), it inhibits differentiation in cultured human PBMCs (Take et al., 2005).

Furthermore, a relationship has been shown between NF-B and Wnt pathway. Because NF-B is activated during inflammation, and chondrocyte DKK1 expression was found to correlate with IL1 and TNF levels (Weng et al., 2009), NF-B could also down-regulate -catenin by inducing DKK1 (Chang et al., 2013). It has also been demonstrated that canonical Wnt signaling stimulates OPG expression in mature osteoblasts leading to the suppression of osteoclast formation (Glass et al., 2005). Finally, it has been indicated that -catenin interacts with p65 of the NF-B signaling pathway, resulting in its activation (Liu et al., 2016).

These properties were originally noted by Yasuda et al. (1998) who made osteoclast-like cells from spleen cells in presence of osteoblasts with IL6, IL11, PTH, and PGE2. However, it is now better understood how Wnt and NF-B pathways are involved in osteoblasts promoting osteoclast differentiation.

### Effect of CFTR on NF-B Pathway

More TRAP+ osteoclasts were observed in CFTR KO mice bone marrow leading to increased osteoclastic bone resorption (Stalvey et al., 2013). CFTR was shown to mediate RANTES expression in airway epithelial cells directly by inserting into the membrane, without its chloride transport function, allowing the activation of NF-B pathway (Estell et al., 2003).

Some direct interactions with CFTR and proteins involved in NF-B signaling have been shown in human bronchial epithelial cells but not yet in osteoclasts. TNFR1 expression is strongly associated with CF due to its interaction with the PDZBD domain of CFTR (Dudez et al., 2008). TNF triggers the translocation of CFTR, TNFR1, and c-Src into the lipid rafts but the recruitment of CFTR and TNFR1 is dependent on protein tyrosine kinase activity and an intact C-terminus domain of CFTR. Intact CFTR contributes to the formation of a TNFR1/c-Src (a proto-oncogene tyrosine-protein kinase) complex in the lipid raft of epithelial cells. This complex stimulates the regulation of gap junction, intracellular communication and IL8 secretion in intact CFTR cells. C-Src is also highly expressed in the ruffled border of osteoclasts (Tanaka et al., 1992) and proven necessary for bone resorption (Roodman, 1999). Its interactions with CFTR has not been shown in osteoclast yet but could explain the decreased resorptive activity of CF osteoclasts (Jourdain et al., 2021).

Cystic fibrosis transmembrane conductance regulator binds also to another compound connecting TNF and NF-B signaling: the TNF receptor-associated death domain protein TRADD (Wang et al., 2016; Figure 4). TRADD binds to SODD, a domain released from TNFR1 throughout TNF binding, and phosphorylates inhibitory protein IB, allowing NF-B to translocate to the nucleus (Pobezinskaya and Liu, 2012). As predicted, TRADD binds to WT-CFTR and G551D CFTR but not to TNR, a variant that has chloride channel function but lacks PDZ binding. Such binding of functional CFTR promotes TRADD degradation and inhibits the ability of TNF to stimulate NF-B activity in human bronchial epithelial cells.

An interaction between CFTR and Krt8 protein has been described in mice (Le Henaff et al., 2016; Figure 4). Genetic deletion of Krt8 in mice expressing F508-del-Cftr resulted in a rescue of the bone phenotype seen in F508-del-Cftr (decreased markers of bone formation and bone mass) in part through the modulation of NF-k-B and Wnt--catenin pathways (Le Henaff et al., 2016). Krt8 interacts with p62, a regulator of NF-B signaling (Janig et al., 2005) and influences osteoblast differentiation by decreasing *RUNX2* and *COL1A1* expression (Le Henaff et al., 2016). However, the study used the F508del mutant, which produces minimal viable CFTR protein, making it inconclusive whether the CFTR-Krt8 interaction is PDZBD- or channel function-dependent. Controversial data of the effect of Inh-172, an inhibitor of CFTR function, has been presented in mouse and humans. In mice, Inh-172 had no significant effect on osteoblast gene expression; whereas, it enhanced RANKL/OPG ratio in human osteoblast (Delion et al., 2016). Therefore, further studies are needed to determine whether CFTR has a direct role on osteoblast differentiation.

Although it has not been shown that NF-B signaling is increased in osteoclasts, other organs, such as lung and pancreas, are affected by the absence of CFTR chloride channel (Al Alam et al., 2010; Cavestro et al., 2010; Gambari et al., 2012; Figure 5), and this, in turn, impacts monocytes and macrophages secretion, leading to the dysregulation of osteoclast differentiation.

Increased MCP-1 levels result in increased osteoclast differentiation and bone resorption. RANKL-induced NFATC1 signaling causes increased expression of MCP-1 cytokine and RANTES chemokine (regulated on activation normal T cell expressed and secreted) which promotes the formation of TRAP-positive multinuclear and bone-resorptive cells (Kim et al., 2005). Studies in other organ systems (lung and pancreas) demonstrated increased MCP-1 and IL-8 secretion as a direct result of CFTR gene mutations (Augarten et al., 2004; Cavestro et al., 2010). This increase in cytokines secretions can be attributed to high levels of NF-B and low levels of IB factor (Tabary et al., 1999). Zaman et al. (2004) proved that a single allelic CFTR mutation was sufficient to increase IL-8 secretion in peripheral blood monocytes in response to lipopolysaccharides (LPS): bacterial endotoxins mainly recognized by the Toll-Like Receptor 4 (TLR4). The reduction of CFTR expression resulted in increased LPS induced cytokine secretion, increased phosphorylation of NF-B, which in turn is a positive regulator of IL-8 expression and decreased IB expression. The mechanism was specified in both CF mice and humans (F508del) macrophage studies (Bruscia et al., 2011). Nave macrophages lacking CFTR had an abnormal TLR4 subcellular localization and trafficking which increased LPS-induced activation of NF-B, MAPK, and IRF-3 pathway. It was also shown that TLR4 was not well degraded in CF macrophages, maybe due to acidification induced by CFTR defect (Shah et al., 2016; Murase et al., 2018).

Endocytosis of certain receptors including TLR4 is disrupted in CF macrophages, due to the acidification generated by a compromised CFTR channel. Velard et al. (2018) showed overexpression of RANK and M-CSFR in monocytes of G551D CF patients, which was partially restored upon treatment with Ivacaftor, a potentiator which increases CFTR-G551D channel opening. This proved a role of the chloride channel function in RANK and M-CSFR expression. Moreover, a recent study reported defective differentiation of CF-F508del human monocytes (PBMC) into osteoclasts. These defects were characterized by a decrease in the number of mature CF-osteoclasts derived from CF PBMCs compared to non-CF; and a higher expression of sphingosine-1-phosphate (S1P) in CF osteoclasts, an important factor in bone formation and density (Jourdain et al., 2021).

To conclude, CFTR mutations resulting in compromised channel function, induces inflammation in both lung and pancreas, which influences osteoclast differentiation (Figure 5). Aberrant chloride channel function influences endocytosis of certain monocyte and macrophage receptors, which may lead to impaired NF-B pathway. Since monocytes and macrophages are progenitors of osteoclasts, impairment in the NF-B signaling may affect osteoclast differentiation and bone resorption.

### Effect of CFTR on Wnt Signaling Through NF-B Pathway and PGE2 Production

Nuclear factor-kappa B signaling is known to inhibit osteogenic differentiation in part by promoting -catenin degradation. F508del mice have been shown to present defective osteoblast differentiation due to increased NF-B signaling and reduced Wnt signaling (Le Henaff et al., 2015). This result was corroborated by a study showing decreased -catenin level in the F508del mouse intestine (Liu et al., 2016).

Cystic fibrosis is associated with prostanoids overproduction, such as PGE2, PGF2, PGF1, and thromboxane B2 in the saliva and urine of CF patients (Jabr et al., 2013). Cyclooxygenase (COX) enzymes are required for the conversion of arachidonic acid into prostaglandins (PGs). COX-2 was described as highly inducible at inflammatory sites, in particular by IL1, and is considered as the main target for NF-B activation (Maier et al., 1990). In lungs, a positive feedback loop exists where PGE2 upregulates both COX-2 expression and p38 MAPK activity during inflammation through cAMP/AMPK signaling (Faour et al., 2008). Both PGE2 and cAMP activator induce COX-2 transcription, by increasing CREB phosphorylation *via* the PKA/p-CREB pathway, with CFTR being a negative regulator (Chen J. et al., 2012). In addition, basal CFTR gene transcription is regulated by intracellular cAMP. Therefore, PGE2 is also responsible of CFTR transcription through the cAMP pathway. Chen J. et al. (2012) suggested that only intact CFTR could be upregulated by PGE2, in turn switching off the PGE2-mediated feedback loop and reducing the inflammatory response. Therefore, it is obvious that a relationship exists between CFTR and PGE2 in lung inflammation.

However, the production of PGE2 by F508del osteoblasts was significantly reduced (Velard et al., 2014). Chikazu et al. (2002) showed that COX2 mRNA and PG production were induced by BMP-2 in osteoblasts *via RUNX2* binding. Although there is presently no direct link described between CFTR and PGE2 in bone cells, an indirect cause may be assumed since *RUNX2* is regulated by Wnt, TGF-- and BMPs-SMAD pathways, which are all downregulated by the NF-B pathway. Low PGE2 production primarily influences Wnt signaling (Goessling et al., 2009) which in turn has an effect on bone formation, stem cell differentiation, inhibition of OPG expression, and indirectly bone resorption *via* upregulation of RANKL in osteoblastic cells (Stalvey et al., 2013; Velard et al., 2014; Delion et al., 2016). The non-canonical Wnt pathway could also be affected by NF-B overexpression in monocytes. Wnt5b has been shown to repress myeloid differentiation (osteoclast progenitors) in the presence of IL-3, and increase it in the presence of GM-CSF, leading to pre-mature progenitor cell exhaustion (de Rezende et al., 2020). The increase of cytokine secretion induced by a defective CFTR could, in association with Wnt5b, over-activate the non-canonical Wnt pathway and dysregulate osteoclast differentiation from myeloid progenitors. Altogether, this suggests that impaired Wnt signaling alters osteoblast and osteoclast differentiation.

### Possible Impacts of CFTR on Bone Resorption

Bone resorption is achieved when osteoclasts attach to the bone matrix and form a bone-resorbing acid compartment by exocytosis of lysosomes. A low pH is necessary for the solubilization of the alkaline salts in bone mineral and the digestion of organic bone matrix (Schlesinger et al., 1997). Acidification is mediated in osteoclast ruffled border by the combined activity of a V-type H+-ATPase pump, which provides the proton force needed to generate a pH gradient, and a chloride channel, that allows a passive chloride transport (Schaller et al., 2004). Vesicles from mature osteoclasts have both pump and channel allowing the resorption, whereas vesicles from osteoclast progenitors (bone marrow cells at different steps of differentiation) have limited acidification with minimal anion permeability. It is only upon exposure to bone that the cells are able to sufficiently express both pump and channel to support acidification (Schlesinger et al., 1997). Although, CFTR has been shown to be expressed both at the membrane and in cytoplasm of osteoclasts and their myeloid progenitors (Shead et al., 2007; Le Henaff et al., 2016), there are currently no studies proving a direct role of CFTR in bone resorption. A new study revealed that a loss of CFTR chloride activity in human osteoclasts, differentiated *in vitro* from human CF-F508del PBMCs, led to a reduced trench-resorption mode (Jourdain et al., 2021). However, the role of CFTR in bone resorption remains poorly understood and needs further investigation.

## Conclusion

Cystic fibrosis transmembrane conductance regulator mutations affecting chloride channel functionality or its possibility to interact with other proteins, impact different signaling pathways: NF-B, Wnt/-catenin, MAPK/ERK, or TGF-. These pathways are interrelated, meaning dysregulation of one has the ability to impact the others. In CF, increased NF-B signaling enhances inflammation *via* monocytes and macrophages, which are both precursors of osteoclasts, which in turn disrupts osteoblast differentiation. Moreover, NF-B prevents Wnt signaling, also resulting in decreased bone formation and increased bone resorption.

The impact of CF on bone resorption has yet to be determined. However, some CF patients develop clinically significant anemia, suggesting that CFTR may regulate hematopoiesis. Furthermore, the hematopoietic system is clearly related to bone as myeloid cells are osteoclast progenitors and allow bone formation through osteoblast differentiation and chondrocyte colonization. Additionally, CFTR plays an important role in chondrocytes, which need chloride ions. Since cartilage formation is the first part of bone formation, the impacts of CFTR mutations on chondrocytes may ultimately affect bone formation, osteoblast differentiation and their reciprocal regulation.

Cystic fibrosis transmembrane conductance regulator mutations perturb many pathways necessary for stem cell differentiation into bone cells. To date, studies have demonstrated the impact of CFTR on mature cells *in vitro* or *in vivo* on mice, fish, rat models. However, nothing has demonstrated the impact of CF on the differentiation process from stem cells to bone cells. iPSCs would be a great means by which to perform these studies, assessing different mutations carried by CF patients. Yet, to be successful, an efficient generation and differentiation method is necessary in order to prevent issues of tumorigenicity and preferential commitment due to the cells epigenetic memory.

## Author Contributions

All authors wrote and edited the manuscript, read and approved the final manuscript.

## Conflict of Interest

The authors declare that the research was conducted in the absence of any commercial or financial relationships that could be construed as a potential conflict of interest. The reviewer JJ declared a shared affiliation with several of the authors, CD and FV, to the handling editor at time of review.
